# Involvement and skepticism towards the JCI Accreditation process among hospital’s four sectors employees: suggestions for cultural change

**DOI:** 10.1186/s13584-021-00507-4

**Published:** 2021-12-14

**Authors:** Shiran Bord, Inna Sass, Gila Hayms, Kobi Moskowitz, Hagar Baruch, Fuad Basis

**Affiliations:** 1 Health Systems Management, The Max Stern Yezreel Valley College, Yezraeel Valley, Israel; 2 JCI Accreditation, Rambam Health Care Campus, Haifa, Israel; 3 Nursing Sector, Rambam Health Care Campus, Haifa, Israel; 4 Hospital Administration, Rambam Health Care Campus, Haifa, Israel; 5 Nursing sector, Rambam Health Care Campus, Haifa, Israel; 6 JCI Accreditation, Technion Faculty of Medicine, Rambam Health Care Campus, Haifa, Israel

## Abstract

**Background:**

Implementing the JCI Accreditation process as an organizational culture may face resistance. However, the skepticism and involvement of different hospital sectors (medical, nursing, paramedical, and administrative/logistic) in the process may vary. Conducting organizational change needs tools to decrease resistance.

**Objectives:**

To investigate the attitudes, cultural norms, and satisfaction of the different sectors regarding the accreditation process, and to suggest ways to integrate the process as part of the organizational culture.

**Materials and methods:**

A cross-sectional survey was conducted among 462 respondents (187 nurses, 95 physicians, 92 administrative, 88 paramedical) at Rambam Health Care Campus, a tertiary hospital in Israel. The hospital employees' attitudes, cultural norms, and satisfaction were assessed. ANOVA tests were used to examine the differences among the different sectors. The association between the satisfaction from the process and the preferred type of training was examined using Pearson's correlation coefficient.

**Results:**

Significant differences were found among the sectors in the scores related to attitude, cultural norms, and satisfaction from the accreditation process (F (3, 456) = 17.95, *p* < 0.001, η^2^ = 0.10). Gabriel post-hoc test revealed significantly lower scores between the medical and paramedical sectors. A positive correlation was found between the degree of satisfaction with the process and the satisfaction with the training type. Frontal education and video demonstrations were rated significantly higher among all 10 training types.

**Conclusions:**

More efforts should be made to increase involvement among physicians and paramedical teams in the accreditation process. Each sector leadership involvement is essential for their involvement too. Early involvement of the Israeli Medical Association in the process might have achieved better physicians’ collaboration. Frontal education and video demonstrations may help decrease skepticism and increase positive attitudes.

## Introduction

The history of the accreditation process began in the United States about a century ago with the establishment of a voluntary body designed to build standards of quality. In 1917, the American College of Surgeons began the implementation of hospital accreditation by setting up "minimum standards for hospitals" [1]. In 1951, the Joint Commission on Accreditation of Healthcare Organizations (JCAHO) was established, and these standards extended to Canada and Australia in the 1960s. In 1998, the international arm of this organization, known as Joint Commission International (JCI), began to develop international evidence-based standards. The international arm of JCI leads various health organizations in implementing the accreditation process in most other countries worldwide [2, 3]. Accordingly, many European countries joined the accreditation process by the mid-1990s, and in the 2000s, the process of accreditation began to spread to Middle (Near) Eastern countries as well, and it can be said that accreditation is now widely accepted in many health systems in many countries around the world, even in less developed ones [4].

The accreditation process strives to improve the quality of medical service patients receive. However, it seems that the implementation of the accreditation process in hospitals may face different challenges that affect its degree of success, and the actual improvement in the quality of care and safety for the patients. One of the most notable challenges in implementing accreditation in hospitals is the full collaboration of hospital staff [5].

Pomey and others examined the implementation of the accreditation process in French hospitals and showed that many times, the difficulty stems from the hospital staff members' distrust of the process, especially in its motives. Hospitals staff members expressed suspicion due to the lack of transparency regarding the use of the accreditation data, and showed concern that the accreditation data would be used for financial sanctions [6]. Similarly, a study conducted at a Lebanese hospital found that despite professionals' recognition of the importance of patient safety, many were skeptical and suspicious of the need to disclose sensitive information [7].

In 2005, JCI's International Standards Program in Israel was implemented by the Clalit Health Maintenance Organization (HMO) hospitals. In 2012, a national program for quality indicators was implemented in hospitals with the obligation of all hospitals in the country to be accredited by the JCI, under the inspection of the Ministry of Health (MOH); that is, the MOH followed the progression of the process to ensure success in the final survey. A study conducted in Israel at the Clalit HMO hospitals examined the training received by the hospital staff and the attitudes of physicians regarding the accreditation process. It was found that physicians from the various accredited hospitals in Israel reported their concern about compromising the autonomy of their profession [8].

Changes in organizations generally tend to cause resistance, especially if the organizational culture is a "routine seeking behavior". A culture of flexibility, cohesion, and trust is needed for implementation of a change and, since it encourages innovation and change, it is negatively correlated with the overall need for a "routine seeking behavior" [9]. Oreg investigated the psychology of the personality associated with resistance to a major change in organizations and found three factors that may influence employee's attitude: job satisfaction, intention to leave the organization, and organizational commitment [10]. According to the organizational literature, there are many reasons to oppose the introduction of changes in organizations. If the change or vision does not match the needs of the employees, threatens their interests, or is deemed unfair, employees will experience anger, outrage, and a desire for retribution [11].

### Background

In June 2016, our hospital, the Rambam Health Care Campus (RHCC), a referral 1056-bed hospital in the north of Israel, passed the first JCI survey, with very few recommended changes, and the surveyors indicated that some of the performance was remarkable. Before the final survey, our hospital passed a mock survey, in which a team of JCI instructors, who are a separate team from the final survey team, conducted a mock test and raised points for correction, thus preparing us for the final test. Our hospital accreditation leadership team consisted of a doctor, a nurse, and an industrial and management engineer, similar to the team of instructors in the mock survey. Our hospital had four sectors: (1) the medical sector (physicians), (2) the nursing sector, (3) the paramedical sector, (4) and the administrative/logistic (operational) sector. The administrative sector consists of a management sector, such as human resources, information system management, and financial department, and a logistic–operational sector consisting of the hospital maintenance sector, including construction, frameworks, and cleaning. The RHCC had over 5000 employees, and the accreditation team had to train the entire hospital staff in the rules and standards of accreditation. Since the process was large, except for the three leaders of the process, it was necessary to recruit and guide employees from other sectors who performed tracers in the inpatient wards. It was very difficult to recruit people from the medical and paramedical sectors. These two sectors had learned little about the new procedures of accreditation, expressed skepticism about the process, tried to evade tasks, and paid less attention to correcting the points our assessment asked for. Therefore, most of the work was done with managers of these sectors and by frontal group training sessions, in which each participant was tested separately in front of their manager and other colleagues. The idea behind the sessions was to put psychological pressure on the subject to meet the expectations of their manager, and the rest of their teammates. Furthermore, this team encountered some lack of cooperation in the mock survey, as some of the hospital staff believed less and some believed more in the importance and impact of the process. Others felt that the project was a burden on the staff in addition to the routine professional tasks they had to meet.

These difficulties, among others, made us feel that there was a gap between the positions of the different professional sectors within the hospital, which led to more or less identification with the process. Further, there was a feeling that some identified with the training methods more than others. Because of these difficulties, we conducted this study to assess where the main difficulties were and find ways to recruit the hospital staff to participate in the process, accept it with less skepticism, and pass the final survey successfully.

### Objectives

Our aim was to investigate the attitudes, cultural norms, and satisfaction of the different sectors of our hospital regarding the accreditation process, and suggest ways to integrate the process as part of the organizational culture.

## Materials and methods

This cross-sectional study took place during preparations to pass the first JCI Accreditation survey and it was performed between the Mock Survey and the Final Survey. We concentrated our concern on four points: 1. Examine the differences in attitudes toward the accreditation process among the four hospital sectors of employment: physicians, nurses, administrative/logistic and paramedical sector (such as technicians, lab staff, social workers, psychologist, and physiotherapist) 2. Check the extent of social and cultural norms and agreement with the hospital accreditation process; 3. Examine the correlation between staff satisfaction with the process and the degree of satisfaction with the training techniques used 4. Learn how the staff rates the 10 different training types in relation to effectiveness and ease.

We conducted a structured questionnaire of 29 statements among hospital employees from all four sectors. The questionnaire consisted of four sections: 1. Demographic and personal characteristics of the participants, such as seniority, professional status, and sector. 2. Participants' attitudes toward the process (statements 1–13), for example: “To what extent do you think the accreditation process will improve the quality of patient care?" 3. Cultural and social norms in the hospital, in relation to the interaction between the staff and the managerial sectors (statements 14–19), for example: “To what extent do you think your superiors think the accreditation is beneficial to the hospital?" 4. Employees' satisfaction with the training program (statements 20–29).

The questionnaire was compiled based on the theory of reasoned action (TRA) questionnaire [12]. Furthermore, we pre-constructed the attitudes, norms and satisfaction questionnaires on the basis of the definitions of these three constructs from the literature [13, 14]. The measurement scale is 1 to 5; where a score of 5 represents a high degree of agreement with the statements and 1 represents disagreement. The satisfaction questions were constructed based on the hospital's experience and were validated by a preliminary sample of 30 people. We calculated the reliability of each questionnaire. For the "attitude" questionnaire, the Cronbach's coefficient α was 0.89, for the "social and cultural norms" had α of 0.74, and the "satisfaction of the training" questionnaire had α of 0.88.

The link to the questionnaire was sent by email to all hospital staff. The questionnaire emphasized that it was anonymous and that the findings would serve as an estimate of project success, promote quality, and for the final survey. Every employee had an organizational e-mail. However, where we had low compliance in completing the questionnaire, as with the operational sector and some of the administrative sector, where their access to a computer or e-mail was low. Thus, we conducted 49 anonymous surveys using the questionnaires sent personally to employees via each sector's manager, and later collected the completed responses from the managers.

Based on a statistical test to calculate the sample size conducted at the beginning of the study, and to maintain a 95% confidence level with a probability of first-rate error of 5%, the sample should include 358 hospital staff. According to the hospital's data as of 2019, there were 5150 employees from all sectors. Our aim was to include 600 respondents, 150 from each sector (medical, nursing, paramedical, and administrative/logistic sectors). However, there were only 462 responders. Many employees do not use the organization mail box and its' content exceeds the full mail capacity, so they did not receive the mail, some do not have access to their mail, or did not activate their mail account, and some did not respond to our mail. Yet, the number of responders was higher than the minimum we needed (462 vs. 358).

The first issue we wanted to examine was whether there was a difference in the attitude, cultural norms, and satisfaction among the four sectors. To compare the averages of the results, we used a one-way analysis of variance (ANOVA) test for the four sectors. If we found a significant difference to reject the null hypothesis (there was no difference between sectors), we planned to go one step down and compare the four sectors in relation to each one of the three above-mentioned parameters, using the Gabriel post-hoc test, as the samples of the different sectors were unequal.

The second issue we wanted to examine was whether there was a positive relationship between the different training methods and the degree of agreement and satisfaction of employees with the accreditation process. We also tried to determine the best training method to engage the hospital staff in the process. We used 10 training techniques to implement the protocols and procedures for the hospital staff. The learning techniques have been adapted according to the specific procedures we wanted the staff to assimilate. These techniques include: (1) frontal teaching presentation and discussion; (2) demonstrative video performed by hospital staff. For example, how to perform the patient identification process; (3) self-learning software in the Rambam-net; (4) divisional accreditation forum, where specific issues related to the specific division are discussed, such as Anesthesia and Surgical Care (ASC) for surgical division; (5) training software with self-assessment; (6) app for procedure and policy in the cell phones (Questions and Answers); (7) multiple choice questions sent to all sectors by SMS, where according to the responder it is possible to know from which sector it was sent. (8) Uncoordinated sudden surveys in the departments; (9) coordinated surveys; and (10) group quiz performed collectively to medical staff. To examine the correlation between the different variables, we used Pearson's correlation coefficient test.

As this was an anonymous questionnaire involving the hospital staff, in consultation with the hospital's IRB, the approval of the Helsinki Committee was not required.

## Results

The least cooperative sector in answering the questionnaire was the residents sector. The sector was examined more often during the mock survey and we examined it more frequently thereafter, during the coordinated and uncoordinated surveys. Perhaps the reason that they responded less to the questionnaire was due to the burden of work and night calls they had, which caused them to check their e-mails only once in a while, instead of more frequently.

A total of 462 respondents answered the survey: 187 nurses (11.2% of 1660), 95 physicians (7.9%% of 1192), 92 from the administrative/logistic sector (7.3% of 1255) and 88 from the paramedical sector (8.4% of 1084). The demographic analysis revealed that approximately 67.2% (324) of the participants were women, quite similar to their percentage among all hospital employees, that is, 64% (Table 1).

**Table 1:** The distribution of the participants according to the different hospital employment sectors

Sector	Total number in the hospital	No. of correspondents	% of Employees in the sector
Physicians	1192	95	7.9
Nurses	1660	187	11.2
Administrative/logistic	1255	92	7.3
Para-medical	1037	88	8.4
Total	5144	462	9

Of the respondents, 51.5% were in a non-managerial position compared to 41.7% in a managerial position at the hospital; 6.8% did not indicate their position. Of those in the managerial positions, there were 194 managers. Further, 43 of 95 physicians (45%), 85 of 190 nurses (45%), 29 of 92 administrative/logistic (31%), and 37 of 88 were from the paramedical sector (42%). The average age was 46.04 years (SD = 10.66, median = 46.00), the average seniority was 19.24 years (SD = 19.13, median = 20.0), and 79.6% work full time positions.

In testing the hypothesis that there was no difference in the average scoring for the attitudes, norms, and satisfaction among the different sectors, one-way ANOVA revealed a significant difference was found among the different sectors (F (3, 456) = 17.95, *p* < 0.001, η^2^ = 0.10) (Table 2).

**Table 2:** Mean and standard deviation of the dependent variables of the study sample (N = 462)

Variables	Mean (scoring)	SD
Attitude towards the accreditation process	3.48	0.70
Norms and culture	3.40	0.50
Satisfaction from the training method	3.50	0.78

Applying the Gabriel post-hoc test, we found that there was a significantly higher scoring in the average of attitudes toward accreditation of the nursing sector than the medical sector (*p* < 0.001), and there is a significantly higher scoring in the average of attitudes of the administrative/logistic sector than the medical sector (*p* < 0.001). However, there was no significant difference in the average of attitudes between the medical and paramedical sectors (*p* > 0.05). Furthermore, we found a higher scoring in the average of attitudes of the nursing sector than the paramedical sector (*p* < 0.001); however, we did not find a significant difference in attitudes between the nursing sector and the administrative/logistic sector (*p* > 0.05). In addition, we found a significantly higher scoring in the attitudes of the administrative/logistic than the paramedical sector (*p* < 0.001) (see Table 3).

**Table 3:** A comparison of each question separately among the four sectors using the One Way ANOVA test

Question	A Paramedical N = 87		B Administrative N = 92		C Nursing N = 186		D Physicians N = 95		F	DF	*p* < 0.05
	Mean	SD	Mean	SD	Mean	SD	Mean	SD			
**Attitude**	**3.22**	**0.75**	**3.63**	**0.65**	**3.68**	**0.62**	**3.19**	**0.64**	**17.95**	**3456**	
1. To what extent were you were involved in the preparations for the accreditation	3.09	0.81	3.59	0.71	3.24	0.68	3.02	0.73	13.14	408	A < B, B > D A < C, C > D A = D, B = C
2. To what extent has the accreditation process improved the quality of care of patients	3.17	0.92	3.61	0.83	3.56	0.72	3.14	0.65	15.81		B > A, C > A B > D, C > D A = D, B = C
3. To what extent does the accreditation process has improved the safety of treatment	3.38	0.68	3.77	0.64	3.91	0.63	3.0	0.81	16.93		B > A, C > A A > D, B > D C > D, B = C
4. To what extent has the accreditation process improved the safety of the employees	3.43	0.83	3.42	0.68	3.57	0.81	3.0	0.57	8.21		A > D, B > D C > D A = B = C
5. To what extent the accreditation process has improved the facilities in your department	3.11	0.84	3.53	0.72	3.71	0.69	3.18	0.59	12.13		A < B, A < C A = D, B = C B > D, C > D
6. To what extent, time and resources invested in the hospital were provided sufficiently	3.08	0.71	3.68	0.81	3.81	0.66	3.25	0.68	10.23		A < B, A < C A = D, B = C C > D
7. To what extent, time and resources for the Accreditation process invested in the hospital were overstated	3.31	0.81	3.49	0.69	4.22	0.61	3.03	0.67	11.23		A = B, A < C A > D, B < C B > D, C > D
8. To what extent written procedures and policies will help you in your work in the future	3.19	0.79	3.75	0.92	3.53	0.66	3.55	0.68	6.32		A < B, A < C A < D B = C = D
9. To what extent the accreditation tracers (surveys) contributed to your work and to the quality of care in hospital	3.28	0.8	3.66	0.58	3.93	0.68	3.21	0.78	12.33		A < B, A < C A = D, B < C B > D, C > D
10. To what extent would you like to take an active part in promoting the accreditation process in your department	3.29	0.72	3.66	0.66	3.67	0.63	3.44	0.66	7.45		A < B, A < C A = D, B = C B = D, C = D
11. To what extent ISBAR or SOAP Can improve the communication between hospital staff	3.09	0.82	3.59	0.69	3.52	0.59	3.04	0.69	13.26		A < B, A < C A = D, B = C B > C, C > D
12. To what extent ISBAR or SOAP Can improve the safety of treatment.	3.18	0.78	3.64	0.64	3.75	0.68	3.5	0.72	5.34		A < B, A < B A < D, B = C B = D, C = D
13. Do you think the accreditation process has influenced the organization culture in the hospital?	3.27	0.79	3.81	0.74	3.72	0.64	2.98	0.75	12.66		A < B, A < C A > D, B = C B > D, C > D
**Norms**	**3.21**	**0.59**	**3.49**	**0.50**	**3.51**	**0.433**	**3.27**	**0.45**	**10.69**	**3449**	
14. To what extent your colleagues think that accreditation is useful to hospital in improving the safety of care	3.29	0.63	3.51	0.55	3.65	0.45	3.27	0.49	5.65		A < B, A < C A = D, B = C B > D, C > D
15. To what extent your superiors think accreditation is useful to the hospital in improving the safety of care	3.12	0.56	3.55	0.57	3.63	0.58	3.38	0.48	7.23		A < B, A, C A < D, B = C B = D, C > D
16. To what extent your superiors expect you to be active in the accreditation process	3.24	0.65	3.43	0.61	3.32	0.41	3.35	0.45	0.78		A = B = C = D
17. To what extent do you think a department that manages the process of accreditation is appraised by hospital management	3.08	0.67	3.58	0.48	3.54	0.52	3.5	0.48	4.76		A < B, A < C A < D B = C = D
18. To what extent your colleagues think that the accreditation process is necessary in light of overburdening the staff	3.19	0.57	3.41	0.61	3.51	0.6	3.15	0.49	7.34		A < B, A < C A = D, B = C B > D, C > D
19. To what extent your superiors think the accreditation process is necessary in light of overburdening the staff	3.23	0.71	3.5	0.62	3.42	0.48	2.98	0.38	5.73		A < B, A < C A > D, B = C B > D, C > D
**Satisfaction from type of training technique**	**3.47**	**0.77**	**3.65**	**0.75**	**3.62**	**0.74**	**3.15**	**0.78**	**9.50**	**3444**	
20. Frontal training in departments	3.33	0.87	3.63	0.82	3.45	0.81	3.15	0.88	7.42		A < B, A = C A = D, B = C B > D, C > D
21. Videos	3.42	0.84	3.68	0.78	3.74	0.83	3.29	0.84	6.82		A = B, A < C A = D, B = C B = C, C > D
22. Presentations in Rambam-net	3.65	0.73	3.71	0.76	3.58	0.92	2.95	0.97	3.23		A = B = C A > D, B = C B > D, C > D
23. Divisional Accreditation Forum	3.54	0.86	3.49	0.65	3.81	0.71	3.21	0.67	4.54		A = B = C A > D, B = C, B > D, C > D
24. Software	3.48	0.79	3.77	0.81	3.67	0.68	3.12	0.87	5.23		A = B = C A > D, B = C B > D, C > D
25. Phone application	3.46	0.68	3.78	0.92	3.43	0.8	3.14	0.81	4.73		A = B = C A > D, B = C, B > D, C > D
26. SMS questionnaires	3.32	0.88	3.54	0.71	3.53	0.72	3.21	0.25	4.56		A < B, A < C A = D, B = C, B > D, C > D
27. Non-coordinated surveys	3.52	0.78	3.61	0.74	3.61	0.75	3.25	0.78	5.21		A = B = C A > D, B = C B > C, C > D
28. Coordinated surveys	3.52	0.83	3.68	0.84	3.79	0.78	3.11	0.78	7.81		A = B = C A > D, B = C B > C, C > D
29. Collective group multiple choice test	3.49	0.75	3.62	0.76	3.58	0.79	3.1	0.88	6.04		A = B = C A > D, B = C B > C, C > D

The signs (<) and (>) indicate a significant (*p* < 0.05) bigger or smaller deference between the compared two sectors while (=) indicate non-significant difference (last Rt. Column). "A" in the rt. column refers the Paramedical sector, "B" in the rt. column refers the Administrative sector, "C" in the rt. column refers the Nursing sector and "D" in the rt. column refers the Medical sector (physicians)

When dealing with cultural norms, applying the one-way ANOVA, we also found a significant difference between the different sectors (F (3, 449) = 10.69, *p* < 0.001, η^2^ = 0.06).

A Gabriel follow-up test found that there was a significantly higher scoring in the average of norms toward the accreditation of the nursing sector than the medical sector (*p* < 0.01). There was also a significantly higher scoring in the average of norms of the administrative/logistic sector than the medical sector and (*p* < 0.05), and there was a significantly higher scoring in the average of norms of the nursing sector than the paramedical sector (*p* < 0.001). There was a significantly higher scoring in the average of norms of the administrative/logistic sector than the paramedical sector (*p* < 0.01). However, there was no significant difference in the average of norms between the medical sector and the paramedical sector (*p* > 0.05), and there was no significant difference in the average of norms between the nursing sector and the administrative/logistic sector (*p* > 0.05).

A one-way ANOVA test also revealed a significant difference in satisfaction with the accreditation process among the different sectors (F (3, 456) = 9.50, *p* < 0.001, η^2^ = 06).

The Gabriel post-hoc test revealed a significantly lower satisfaction of the medical sector toward the accreditation compared to the nursing sector (*p* < 0.001), and there was a significantly lower satisfaction between the medical sector and the administrative/logistic sector (*p* < 0.001). We found that there was no significant difference in the satisfaction between the nursing sector and the paramedical sector (*p* > 0.05), and there was no significant difference in the satisfaction between the nursing sector and the administrative/logistic sector (*p* > 0.05). There was also no significant difference in satisfaction between the administrative/logistic sector and the paramedical sector (*p* > 0.05). However, there was a significantly higher scoring in the satisfaction of the paramedical sector compared to the medical sector (*p* < 0.05) (see Table 3).

We went one step further and performed an ANOVA test for the 29 questions we calculated the F value of all the sectors and with the help of the Gabriel post hoc test we made a comparison between the 4 sectors for each question and question (Table 3). The results are summarized in Table 3 in the last column on the right. The sign (>) and (<) indicate a significant statistical difference, while (=) indicates a statistically insignificant difference.

The sector mostly involved in both patients' safety and departments' facilities management safety (FMS section) is the nursing sector. The operational/administrative sector is very involved in the FMS section. The medical sector is mainly examined in the quality of the medical record, as is the paramedical sector. When we delve into the answers to the specific questions in Table 3, in the "Attitude" questionnaire, we see that in question number 3 (To what extent does the accreditation process has improved the safety of treatment), both the nursing sector and the operational/administrative sector gave a very high score, while it can be seen that the nursing sector gave the maximum score for question number 7 (To what extent, time and resources invested in the hospital were overstated). This may be partially due to the overburden in the preparations for accreditation that falls on the nursing staff. This sector is responsible for the safety of medical care, extensive documentation in the medical record, as well as the safety of the facilities in the wards. In other words, they expected to deal with other sectors duties. This may explain them complain about the process (see “Discussion” section below).

At the same time, the nursing sector gave a high score (3.91) to question number 9 (To what extent the accreditation tracers (surveyors) contribute to your work and may improve the quality of care in hospital), which reflects high involvement of the nursing sector in the process.

When we look at the "Norms" questionnaire that relates more to the hospital's corporate culture, it is interesting to see that respondents gave themselves a higher score than they think their managers and colleagues think about the same point. For example, comparing question number 15 (To what extent your superiors think accreditation is useful to the hospital in improving the safety of care), to question number 3 (To what extent the accreditation process has improved the safety of treatment) shows that their personal scoring is higher than that they attribute to their managers. The same is true in comparing the score of question 3 to question 14.

In a questionnaire that deals with the best way of training, the nursing sector gave the highest score to question number 25 relating to the app available online on the mobile phone. Since most of the questions asked by the surveyors relate to the nursing sector, and as it is acceptable to use the app while they are examined, it probably contributed to the high score given by the nursing sector. The medical sector gave the highest score to videos that lasted between 5 and 10 min and addressed the most important part of the International Patient Safety Goals (IPSG), which must be passed with a score of 100. It seems that physicians have no time or patients to read material that can be summarized in a short video.

Testing the hypothesis that there was no relationship between the degree of agreement with the accreditation process (cultural norms), and the satisfaction of employees with the various types of training, we used the Pearson correlation coefficient. It revealed a significant positive relationship between the variables (rp = 0.37, *p* < 0.001), that is, the more positive the degree of consent (norms), the higher the employee's satisfaction with the various types of training (see Table 4).

**Table 4:** Average and standard deviation of the different types of training and satisfaction from them

Training method	Mean	SD
Frontal teaching and questionnaire	3.89	0.94
Demonstrative videos	3.83	1.05
Self-learning presentation at the hospital internet site	3.20	1.17
Divisional Accreditation Forum	3.36	1.17
Training software	3.45	1.16
App for procedures and policy	3.37	1.25
Questionnaires by SMS	3.19	1.25
Uncoordinated sudden exams	3.45	1.15
Coordinated exams	3.59	1.09
Group test for doctors in the wards	3.64	1.10

By applying the one-way ANOVA test, we found a significant difference in the scoring between the different techniques of education (F (9, 452) = 20.962, *p* < 0.001, η^2^ = 0.07). Using the Gabriel post-hoc test, we found that the frontal and the video education techniques were significantly more favorable to the staff (*p* < 0.001 and *p* < 0.01 respectively). However, there was no difference between both of these techniques (*p* = 0.372).

## Discussion

In Israel, the MOH imposed the JCI Accreditation process on all public hospitals in the country. Hospital managers did not get an extra budget to cover the costs of the process. We also noticed that in our hospital, the process progressed at a slow pace, and as the date of the survey approached, employees became more involved and more stressed. The nursing sector was more cooperative; however, like others, they complained that they could not cope with the documentation of certain procedures due to the need for further nursing staff [15]. In parallel, in the Clalit HMO, which supplied medical insurance to about 51.7% of the citizens (2019–2020), with eight hospitals in the country, physicians boycotted the process because of the above-mentioned claims. However, these hospitals passed the surveys three times until now without their partial participation [16].

So, why was there a difference between the attitudes, norms, and satisfaction of the nursing and the administrative/logistic sectors and those of the medical and paramedical sectors?

Our hypothesis is consistent with Mannion's hypothesis that both the physicians and the paramedical sectors work as individuals; each caregiver deals with one patient at a time, rather than dealing with all patients in the department, as nurses do. Furthermore, these two sectors are used to give instructions to other caregivers; therefore, it might be that they have difficulties in receiving non-professional orders [17]. Further investigation of this point may be needed. Moreover, in informal conversations, many members of the Israeli Medical Association expressed their objection to the appraisal of doctors by their superiors and their fear that this could be used against them in professional advancement or lawsuits. In discussions we had with the physicians' committee at the hospital and with the head of the Association of Government Hospitals in the Medical Association, the union's position is firmly against the evaluation of physicians. Their contention that a written medical evaluation can be used against a physician's promotion if his manager decides to undermine the physician's image for unjustified personal reasons. Further, if the physician were sued for negligence, the court could seek a personal case, and the prosecution could use what was written about it unjustly against him.

Since about 41% of responders had managerial roles, it was of interest to categorize managerial roles according to the different sectors and examine their impact on the results, and whether their participation might have biased the results. Based on this analysis, we observed that the sector results of the 42% who were managers in the paramedical sector were no longer positive. However, 45% of the nursing sector respondents had managerial positions, and the sector’s score was relatively high when only these managers were considered. Similarly, the only 31% who were managers in the administrative/logistics sector were highly satisfied with the process, whereas the attitude of the 45% who were managers in the physician sector was not so positive. The results may also reinforce our findings that the managerial arm in the medical sector was skeptical of the process. Together, these findings indicate that it cannot be assumed that the scales might have been tipped in the positive direction because 41.7% of the respondents were managers.

In this context, a large study conducted among 30 hospitals in Denmark showed that hospital managers had the highest positive attitudes toward the Danish accreditation process, the nurses were in second place, and physicians were in the last place. The para-clinical and administrative sectors were not involved in the study, as was the case in ours [13]. However, another study conducted in Iran at some medical centers found that the attitudes of the nursing and managerial sectors were skeptical of the local accreditation process, while the paramedical sector was even more skeptical of the process. In the discussion, the authors noted that, in their opinion, the skepticism of all sectors stemmed from the heavy workload required of managers and other staff to do [18]. It seems that the behavior of the managers themselves, too, may differ from one hospital to another, possibly according to the degree to which their superiors allow them to participate or be involved in the decision-making process [19].

Another point that we examined was the attitude of different sectors toward the educational method utilized to prepare for the survey (Table 4). We found that the sectors that were satisfied and engaged with the process showed more satisfaction with the various training methods. We found that techniques that need more effort, and are time-consuming, such as teaching software and some of the reading materials, were less educational, effective, or desired. Participating in interactive teaching and discussion was deemed significantly more educational and desired [20], as was watching a short video performed by hospital actors. This point is worth considering in future preparations.

Unfortunately, the hospitals did not receive resources or sufficient time to learn or prepare for the assessment. All training was conducted in all sectors during daily work in coordination with the department heads. Preparations for the first survey took more than two years. The training was customized for each sector according to its field of practice. The hospital staff did not receive training beyond working hours. The frontal trainings were conducted in division forums at prearranged hours in the hospital hall. Except for the surprise tests, all the tutorials and tests were conducted in coordination with the department heads.

Some articles in the literature have discussed hospital staff's attitudes toward the accreditation process. Most of these articles dealt with the nursing sector, a small part with the medical sector, and even fewer with the paramedical sector. We have not found in the literature a reference to the administrative/logistics sector, which makes up about 25% of our hospital staff. In a review of the literature, Andri et al. found seven articles dealing with nurses' perceptions of the JCI Accreditation process in different countries, and showed that the increased knowledge and perception of nurses about patient safety culture contributed to improving the quality of hospital services [21]. Another study showed that a hierarchical quality culture declined among physicians as time passed following accreditation, whereas it increased among nursing staff [22]. Furthermore, according to Mannion and Davies, the main difficulty in assessing the relationship between organizational culture and accreditation is that hospitals are a dynamic cultural mosaic composed of many complex subgroups, some of which hold different assumptions, values, beliefs, and behaviors. For example, physicians tend to focus on patients as individuals rather than as groups and act based on exact scientific approaches; that is, their primary goal is to diagnose and cure diseases. These differences have important implications for the implementation of changes or the maintenance of the status quo, the delaying of organizational changes, and, as a result, the implementation of accreditation in health organizations [17]. Further, physicians tend to adopt clinical guidelines only after they are evidence-based; therefore, they contend that accreditation has not been proven to improve treatment outcomes and mortality rates [23]. However, in a review of the literature by Hinchcliff et al. dealing with the accreditation process, they found that, due to the limitations of the studies in the literature, it is not prudent to make strong claims about the effectiveness or non-effectiveness of health service accreditation [24]. Therefore, in our opinion, this skepticism about the process does not necessarily mean that the accreditation process is ineffective in improving morbidity and mortality in hospitals.

Our study was conducted during the period between the mock survey and the final survey. The trainings and the assessments made between the two periods led to a better implementation of the procedures and of the working methods, to improving the safety of the treatment and drawing lessons from misses and near misses, as part of the organizational culture, and to great success in the final survey. Continuous training all along pre- and post-survey periods, not just before the survey, is recommended, and the process should be adopted throughout the period between surveys as a way of work.

## Conclusions and implications of this study

To implement a change, it would be easier if the hospital leadership showed commitment to the process without hesitation. A lot of information must be shared and persuasion have to be done [25]. Furthermore, the MOH should avoid relying on the normative pressure of "new product" introduction, as has been done with hospital managers. To reduce resistance, there may be a need to involve employees in decision making, lead a change with key figures, and give hospital managers a budget to prepare the hospital for the survey. The involvement of employees in the process, as we did after drawing the conclusions from this study, was proven to decrease resistance, increase satisfaction, and lead to more cooperation in conducting a change by organizations, as Levy suggested in his book.

In the context of what Levy wrote, the Medical Association was not a partner in applying the accreditation from the early stages, and the process was imposed on the hospitals, first by the management of the Clalit HMO and later by the MOH. The Israeli Medical Association objected to providing written feedback to senior physicians on an annual basis for fear of harming physicians' progress for personal reasons of their managers, and for fear of negative use of written feedback against physicians during a lawsuit.

At our hospital, the doctors cooperated, however the local doctors' council objected to this point and we received a comment for correction in the assessment. The medical sector argues that what is appropriate for the American system is not necessarily appropriate for the system in Israel. Some even proposed the establishment of an Israeli Accreditation process, as several countries around the world have done. The authors believe that a solution to this issue must be in collaboration with the Ministry of Health, the Medical Association and the JCI in order to reach a win–win situation and achieve cooperation of the medical sector. Giving feedback to senior physicians resulted in Clalit HMO physicians boycotting the process and yet they were accredited. Therefore, the authors believe, out of familiarity with all JCI standards, that the evaluation of senior physicians is not crucial in achieving safe patient care, and in our view, there is room for compromise between the three parties, thus achieving better physicians' collaboration.

## Limitations of the study

The participation of the residents in the study was poor. Although some studies have shown that the attitude among residents toward the process is low due to their large workload and fewer opportunities to learn about the process  [26], it would be interesting to see what they think about the process as a younger generation. We also had difficulty in having the paramedical sector participate in the study. Further effort might have been needed in this regard.
